# Armed Conflict in Central America and Immigrant Health in the United States

**DOI:** 10.29024/aogh.2373

**Published:** 2018-11-05

**Authors:** Jeremy C. Green, Rhonda BeLue, Eric Adjei Boakye, Esther Choi, Michael G. Vaughn

**Affiliations:** 1 Saint Louis University, Department of Health Management and Policy, US; 2 Saint Louis University, Center for Outcomes Research, US; 3 University of Maryland, Department of Behavioral and Community Health, US; 4 Saint Louis University, School of Social Work, US

## Abstract

**Background:**

While many researchers document the immediate and localized health effects of armed conflicts on combatants are well documented in the literature, less is known about the effects of armed conflict on individuals who have subsequently migrated elsewhere.

**Objective:**

This study aims to estimate associations between pre-migration armed conflict in Central America and post-migration health in the United States.

**Methods:**

We created a new dataset that combines information on armed conflicts in Central America and immigrant health in the United States. We used ordered probit regressions to estimate age-adjusted associations between pre-migration armed conflict and post-migration health.

**Findings:**

The study sample of Central American immigrants included 15,563 females and 16,236 males between the ages 15 and 69. The mean age was 37.2 years (standard deviation, 11.6 years) for females and 35.5 years (standard deviation, 11.2 years) for males. After adjusting for age, pre-migration armed conflict was associated with a 8.6 percentage point decrease in excellent health for females (95% confidence interval, 6.0 to 11.1), and a 7.3 percentage point decrease for males (95% confidence interval, 4.0 to 10.7). Each decade of pre-migration armed conflict was associated a 2.9-percentage point decrease in excellent health for females (95% confidence interval, 2.0 to 3.8) and a 1.6-percentage point decrease for males (95% confidence interval, 0.6 to 2.6). For those individuals exposed to armed conflict, each decade since the most recent armed conflict was associated with a 1.5 percentage point increase in excellent health for females (95% confidence interval, 0.4 to 2.5). For males, the average marginal effect of decades since last conflict was not statistically significant (95% confidence interval, –0.001 to 0.002).

**Conclusions:**

Pre-migration armed conflict in Central America is associated with decreases in excellent post-migration health in the United States. The effects of armed conflict are cumulative and fade over time for females.

The health effects of armed conflict are a relatively understudied topic within the public health and social science literatures [1,2,3]. In particular, research is needed on health effects of armed conflicts which can include forced migration or exile [4,5], disrupted or debilitated health care services [6], lack of access to education [7], and even lack of access to electricity and water [8]. The population health effects of armed conflicts can be difficult to document for various reasons [9], including the forced migration that can result from armed conflict [10,11]. A subset of the literature on the health effects of armed conflict compares the outcomes of individuals who migrate for reasons related to conflict, as compared to those who migrate for other reasons [12,13,14].

Yun and colleagues [14: p266] analyzed cross-sectional data from the Princeton New Immigrant Survey to examine health outcomes of refugees and immigrants to the United States. Individuals in the study reported disturbing pre-migration experiences including persecution, incarceration, physical punishment, confiscation of property, unemployment, property damage, and threats. Post-migration health outcomes were similarly problematic and included depression, pain, poor self-rated health, and declining health. A systematic review of the literature described similar pre-migration exposures to armed conflict and post-migration health problems among immigrants to the United States [15]. Armed conflicts have long-term effects on health outcomes [16], including physical outcomes such as life expectancy [17], and mental health outcomes such as anxiety, depression, and mood disorders [18,19]. Post-migration health outcomes might also reflect lack of resources following armed conflicts including economic insecurity [20], disrupted social networks [21], and inadequate housing [22].

The health effects of armed conflict are an increasingly important research topic as increasing numbers of individuals live in conflict or post-conflict areas worldwide [23]. One noteworthy shortcoming in the literature is the relative lack of evidence to date on links between pre-migration armed conflict to post-migration population health outcomes. Our study expands the literature on pre-migration armed conflict and post-migration health outcomes by quantifying associations between pre-migration armed conflict in Central America and post-migration health outcomes in the United States. Understanding the health of immigrants in the United States will become increasingly important, as more immigrants settle in the United States than in any other country [24]. We studied Central America because of the high rates of armed conflict and violence in this region [25,26]. We study long-term effects of armed conflict in displaced populations, which can persist over time [27] and be detected for decades following the conflicts [28,29].

Our study is motivated by conceptual frameworks that describe the wide-ranging public health consequences of armed conflict [30], including long-term effects on the life-course of noncombatant and civilian populations [31]. Additional conceptual motivation for our empirical work comes from a literature on immigrant health over the life-course, including cumulative disadvantage that can result from pre-migration and early life experiences [32]. Our research contributes to an empirical literature that links pre-migration violence to post-migration health outcomes [33,34,35]. The current study is most closely related to Joly and Wheaton [39: p89], who relate pre-migration armed conflict in immigrant sending countries to post-migration health anxiety and depression in Canadian immigrant populations. Our primary research aims are to estimate effects and cumulative effects of pre-migration armed conflict on post-migration health status. In addition, we aim to test whether the health effects of armed conflict fade over time, and whether the effects of armed conflict vary by gender.

## Methods

To estimate associations between pre-migration exposure to armed conflict in Central America and post-migration health outcomes of Central American immigrants in the United States, we created a new dataset that combines information on country-by-year variation in pre-migration armed conflict exposure, with individual-level post-migration health outcomes and relevant covariates. The study sample included individuals born in Belize, Costa Rica, El Salvador, Guatemala, Honduras, Nicaragua, and Panama who subsequently migrated to the United States. We analyzed this new dataset using descriptive and inferential statistics to study the effects of pre-migration armed conflict and post-migration health outcomes. The Saint Louis University institutional review board determined that the research did not constitute human subjects research, and was therefore exempt from further review.

### Data Sources

We combined information from two data sources to create the analytical file for this study. To start, we analyzed country-by-year variation in armed conflicts using the Major Episodes of Political Violence file from 1948 to 2015 [36]. The file is publically available, updated annually, and includes data on armed conflicts around the world. The Major Episodes of Political Violence file is one of the data sources available as part of the Armed Conflict and Intervention series of files. The Armed Conflict and Intervention files are collected and maintained by the Integrated Network for Societal Conflict Research at the Center for Systemic Peace.

Next, we analyzed individual-level variation in post-migration health outcomes using repeated cross-sectional data from the Integrated Public Use Microdata Series adult sample from 1996 to 2015 [37]. The Integrated Public Use Microdata Series combines information from multiple sources across different years to facilitate the analysis of multiple years of individual-level data. Within the Integrated Public Use Microdata Series, we analyzed data from the Current Population Survey, and the Annual Social and Economic Supplement. The Annual Social and Economic Supplement is an annual survey completed in March of each year, and includes data on health status. These data are collected by the United States Bureau of Labor Statistics and the United States Census Bureau and gathered from in-person and telephone interviews. For our analysis, we included demographic data on individual country of birth, year of immigration to the United States, individual age in years, and male or female gender. In addition, we included the sampling weights. The sampling weights can help to correct estimates and standard errors for elements of the survey design, including non-response and an oversampling of Hispanic individuals.

### Study Variables

The dependent variable for our analysis was individual-level, self-reported health status measured on a five-category Likert scale of excellent health, very good health, good health, fair health, and poor health. Self-reported health status was collected as part of the Annual Social and Economic Supplement survey included in the Integrated Public Use Microdata Series. The survey question was worded as follows: “Would you say your health in general is excellent, very good, good, fair, or poor?” The key independent variables for our analysis were individual-level, pre-migration measures of exposure to armed conflict in Central America, during or after the year of birth and before or during the year of immigration to the United States, for each individual in the dataset. To create the pre-migration measures of exposure to armed conflict, we combined country by year data from the Major Episodes of Political Violence with repeated cross-sections of individual data from the Current Population Survey. We created three different pre-migration measures of armed conflict exposure – a dichotomous variable for any pre-migration armed conflict exposure, a continuous variable for the number of years of pre-migration armed conflict exposure and, within the sample of individuals with any pre-migration armed conflict exposure, a continuous variable for the number of years since the most recent year of pre-migration armed conflict exposure.

To create the pre-migration measures of armed conflict exposure, we first extracted the relevant information on armed conflicts in Central America from the Major Episodes of Political Violence file and then coded these events in the Current Population Survey over the life-course of each individual in the microdata. In the Major Episodes of Political Violence file, we selected the country by year observations that had any indicators of armed conflict. For each year and country of armed conflict, we created an indicator for pre-migration exposure to armed conflict that flagged each individual in the microdata who was born in the country of the armed conflict, before or during the armed conflict, and subsequently migrated to the United States during or after the armed conflict.

We summed these armed conflict indicators within each individual in the microdata, to create the continuous variable measuring number of years of pre-migration armed conflict exposure during or after the year of birth and before or during the year of migration to the United States. We then subset the study sample to include on those individuals with at least one year of pre-migration armed conflict exposure, to create the number of years since the most recent year of pre-migration armed conflict exposure. To create this variable, we subtracted the year of the most recent pre-migration armed conflict exposure from the survey year, for each individual separately. To facilitate the interpretation of regression analyses, we divided the years of pre-migration armed conflict, and the years since the most recent pre-migration armed conflict, by 10 to create variables for decades of pre-migration armed conflict and decades since the most recent pre-migration armed conflict.

### Statistical Analysis

The statistical analysis of the study included three main steps – describing the prevalence of pre-migration exposure to armed conflict among individuals included in the study sample, describing the unadjusted associations between pre-migration armed conflict exposure and the distribution of post-migration self-reported health status, and estimating age-adjusted associations between the pre-migration conflict exposure and the post-migration health outcome. For each step of the statistical analysis, we stratified the study sample by gender, to examine the exposure and associations with outcomes for males and females separately. To describe the prevalence of pre-migration armed conflict exposure in the study sample, we examined the frequency of one or more years of pre-migration armed conflict for each country of birth and gender included in the study sample. To describe the unadjusted associations between pre-migration armed conflict exposure and the distribution of post-migration self-reported health status, we examined cross-tabulations between the pre-migration exposure and the post-migration outcomes for each gender separately and used chi-square tests to quantify the statistical significance of the association between these two variables of interest, and considered *P* values less than 0.05 from two-sided hypothesis tests to be statistically significantly different from zero.

To estimate unadjusted associations and age-adjusted associations between pre-migration armed conflict exposures and the post-migration health outcomes, we used a regression-based design. The regression-based design consisted of two steps – fitting the regression models, and generating the predictions [38]. First, we fit multivariate, ordered probit regressions of the five categories of health status on each of the measures of pre-migration armed conflict exposures, separately. In the adjusted analyses, we added control variables for age, age squared, and age cubed in multivariable specifications of the baseline models. Second, we examined the marginal effects of each of the measures of pre-migration armed conflict exposure on each of the five categories of health status. In the adjusted analyses, we conditioned the marginal effects on the observed distributions of age, age squared, and age cubed. For the dichotomous independent variable of any pre-migration exposure to armed conflict, we examined the incremental marginal effects. For the continuous independent variables for decades of pre-migration exposure to armed conflict, and for decades since the most recent pre-migration exposure to armed conflict, we examined the average marginal effects.

We used the delta method to compute 95% confidence intervals from two-sided hypothesis tests [39]. Marginal effect estimates and corresponding confidence intervals were corrected for aspects of the sampling design of the Annual Social and Economic Supplement survey, using weights from the survey for the inverse probability that each observation is included in the study. These sampling weights incorporate various aspects of the survey design into inferential statistics, including corrections for non-response and the oversampling of Hispanic individuals in the Annual Social and Economic Supplement surveys fielded during March of each year. Statistical analyses were carried out in Stata software version 14, manufactured by StataCorp [40].

## Results

The study sample included a total of 15,563 females and 16,236 males between the ages of 15 and 69 at the time of the health outcomes survey. Thirteen individuals were excluded from the study sample due to missing data on year of immigration. The mean age of individuals in the study sample was 37.2 years (standard deviation, 11.6 years) for females and 35.5 years (standard deviation, 11.2 years) for males. The following sections present results of the descriptive analysis, which finds statistically significant associations between pre-migration armed conflict and post-migration health status and the regression analysis, which finds that the age-adjusted health effects of armed conflict accumulate over time for males and females, and fade over time for females.

### Descriptive Analysis

Table 1 reports the prevalence of pre-migration armed conflict experienced by Central American immigrants, stratified by country of birth and by gender. The prevalence of pre-migration armed conflict ranged from a low of 0% in Belize to a high of 99.1% in Guatemala among females, and from a low of 0% in Belize to a high of 99.0% in Guatemala among males. For Central American immigrants across all countries of birth included in the study sample, the overall prevalence of pre-migration armed conflict was 90.9% for females and 92.4% for males.

**Table 1:** Pre-migration Armed Conflict for Central American Immigrants^a^.

Country of Birth	Armed Conflict, No. (%)	No Armed Conflict, No. (%)	Total, No. (%)

**Females (n = 15563)**			

Belize	0 (0)	213 (100)	213 (100)
Costa Rica	81 (17.12)	392 (82.88)	473 (100)
El Salvador	6733 (97.23)	192 (2.773)	6925 (100)
Guatemala	3542 (99.05)	34 (0.951)	3576 (100)
Honduras	2219 (95.03)	116 (4.968)	2335 (100)
Nicaragua	1260 (95.74)	56 (4.255)	1316 (100)
Panama	309 (42.62)	416 (57.38)	725 (100)
Total	14144 (90.88)	1419 (9.118)	15563 (100)
**Males (n = 16236)**			

Belize	0 (0)	136 (100)	136 (100)
Costa Rica	64 (15.09)	360 (84.91)	424 (100)
El Salvador	6781 (96.83)	222 (3.170)	7003 (100)
Guatemala	4696 (99.03)	46 (0.970)	4742 (100)
Honduras	2267 (94.97)	120 (5.027)	2387 (100)
Nicaragua	1055 (95.13)	54 (4.869)	1109 (100)
Panama	131 (30.11)	304 (69.89)	435 (100)
Total	14994 (92.35)	1242 (7.650)	16236 (100)

^a^ Table entries are counts and frequencies of pre-migration armed conflict. For each individual, pre-migration armed conflict includes any armed conflict that occurred in their country of birth, during or after their year of birth, and before or during their year of immigration to the United States.

Tables 2 and 3 report the mean number of years of pre-migration armed conflict and the number of years since the most recent pre-migration armed conflict, respectively. The results in Table 2 show that the mean number of years of pre-migration armed conflict ranged from a low of 0 years in Belize to a high of 17.4 years in Guatemala (standard deviation, 7.6 years) for females, and from a low of 0 years in Belize to a high of 16.9 years in Guatemala (standard deviation, 7.5 years) for males. Overall, the mean number of years of pre-migration armed conflict was 10.9 years for females (standard deviation, 7.5 years) and 11.5 years for males (standard deviation, 7.4 years).

**Table 2:** Years of Pre-migration Armed Conflict for Central American Immigrants^a^.

Country of Birth	Years of Armed Conflict, Mean (SD)	

Gender	Females (n = 15563)	Males (n = 16236)

Belize	0 (0)	0 (0)
Costa Rica	0.218 (0.514)	0.179 (0.452)
El Salvador	9.255 (4.902)	9.363 (4.817)
Guatemala	17.37 (7.583)	16.89 (7.453)
Honduras	13.89 (7.153)	13.34 (7.068)
Nicaragua	7.846 (4.119)	7.826 (3.965)
Panama	0.426 (0.495)	0.301 (0.459)
Total	10.88 (7.495)	11.48 (7.372)

Abbreviation: SD, standard deviation.

^a^ Table entries are means and standard deviations for the number of years of pre-migration armed conflict. For each individual, the number of years of pre-migration armed conflict includes years of armed conflict that occurred in their country of birth, during or after their year of birth, and before or during their year of immigration to the United States.

**Table 3:** Years Since Pre-migration Armed Conflict for Central American Immigrants^a^.

Country of Birth	Years Since Last Armed Conflict, Mean (SD)	

Gender	Females (n = 14144)	Males (n = 14994)

Belize	--^b^	--^b^
Costa Rica	50.89 (5.422)	51.17 (5.545)
El Salvador	19.15 (6.988)	18.65 (6.680)
Guatemala	17.16 (7.752)	16.11 (7.085)
Honduras	19.63 (6.725)	18.99 (5.992)
Nicaragua	19.80 (8.815)	19.49 (8.121)
Panama	18.19 (5.300)	18.40 (5.280)
Total	18.94 (7.739)	18.10 (7.255)

Abbreviation: SD, standard deviation. ^a^ Table entries are means and standard deviations for the number of years since the last pre-migration armed conflict. For each individual, the last pre-migration armed conflict occurred in their country of birth, during or after their year of birth, and before or during their year of immigration to the United States. ^b^ There were no armed conflicts in Belize during the study period.

The results in Table 3 show, in the subsample of individuals with at least one year of pre-migration armed conflict, that the mean number of years since the last pre-migration armed conflict ranged from a low of 17.2 years in Guatemala (standard deviation, 7.8 years) to a high of 50.9 years in Costa Rica (standard deviation, 5.4 years) for females and from a low of 16.1 years in Guatemala (standard deviation, 7.1 years) to a high of 51.2 years in Costa Rica (standard deviation, 5.5 years) for males. Table 4 reports the health status of Central American immigrants in the United States, stratified by pre-migration armed conflict exposure and by gender. The distribution of health outcomes was statistically significantly associated with pre-migration armed conflict exposure, for both females and males.

**Table 4:** Central American Immigrant Health, by Pre-migration Armed Conflict^a^.

Health	Armed Conflict, No. (%)	No Armed Conflict, No. (%)	*P* Value^b^

**Females (n = 15563)**			

Excellent	3338 (23.60)	472 (33.26)	<.001
Very good	4650 (32.88)	481 (33.90)	
Good	4547 (32.15)	343 (24.17)	
Fair	1248 (8.824)	85 (5.990)	
Poor	361 (2.552)	38 (2.678)	
Total	14144 (100)	1419 (100)	
**Males (n = 16236)**			

Excellent	4015 (26.78)	473 (38.08)	<.001
Very good	5161 (34.42)	420 (33.82)	
Good	4594 (30.64)	275 (22.14)	
Fair	964 (6.429)	52 (4.187)	
Poor	260 (1.734)	22 (1.771)	
Total	14994 (100.00)	1242 (100)	

^a^ Table entries are counts and frequencies of individuals in the study sample by pre-migration armed conflict and health status. For each individual, pre-migration armed conflict includes any armed conflict that occurred in their country of birth, during or after their year of birth, and before or during their year of immigration to the United States. ^b^ *P* values are from chi-square tests.

### Regression Analysis

In Tables 5, 6, 7, we present the results of the regression analysis on the post-migration health effects of pre-migration armed conflict. Within each table of regression results, the top panel reports the unadjusted associations between pre-migration armed conflict and post-migration health status, and the bottom panel reports age-adjusted associations between pre-migration armed conflict and post-migration health status. Within each panel of the regression results, the first column reports results for females, and the second column reports results for males.

**Table 5:** Pre-migration Armed Conflict and Central American Immigrant Health^a^.

Health	Incremental Effect^b^ (95% Confidence Interval^c^)	

**Unadjusted**		

**Gender**	**Females (n = 15563)**	**Males (n = 16236)**

Excellent	–0.0892 (–0.115, –0.0637)	–0.0824 (–0.116, –0.0488)
Very good	–0.0118 (–0.0137, –0.00982)	–0.00461 (–0.00663, –0.00259)
Good	0.0562 (0.0402, 0.0722)	0.0568 (0.0341, 0.0795)
Fair	0.0321 (0.0241, 0.0400)	0.0218 (0.0142, 0.0293)
Poor	0.0127 (0.00974, 0.0157)	0.00843 (0.00573, 0.0111)
**Adjusted^d^**		

**Gender**	**Females (n = 15563)**	**Males (n = 16236)**

Excellent	–0.0856 (–0.111, –0.0604)	–0.0732 (–0.107, –0.0396)
Very good	–0.0113 (–0.0133, –0.00944)	–0.00466 (–0.00626, –0.00307)
Good	0.0541 (0.0382, 0.0699)	0.0507 (0.0279, 0.0735)
Fair	0.0309 (0.0230, 0.0388)	0.0197 (0.0119, 0.0276)
Poor	0.0120 (0.00907, 0.0149)	0.00748 (0.00474, 0.0102)

^a^ Table entries are incremental effects and 95% confidence intervals for the associations between pre-migration armed conflict and post-migration health status. For each individual, pre-migration armed conflict includes any armed conflict that occurred in their country of birth, during or after their year of birth, and before or during their year of immigration to the United States. Incremental effects and 95% confidence intervals are estimated using sample weights. ^b^ Incremental effects are from ordered probit regressions of the distribution of health status on pre-migration armed conflict. ^c^ 95% confidence intervals are computed using the delta method. ^d^ Adjusted incremental effects and 95% confidence intervals are conditioned on the observed distributions of age, age-squared, and age-cubed.

**Table 6:** Decades of Pre-migration Armed Conflict and Central American Immigrant Health^a^.

Health	Average Marginal Effect^b^ (95% Confidence Interval^c^)	

**Unadjusted**		

**Gender**	**Females (n = 15563)**	**Males (n = 16236)**

Excellent	–0.0373 (–0.0460, –0.0286)	–0.0293 (–0.0389, –0.0197)
Very good	–0.00895 (–0.0111, –0.00681)	–0.00440 (–0.00592, –0.00287)
Good	0.0234 (0.0179, 0.0289)	0.0206 (0.0139, 0.0274)
Fair	0.0158 (0.0121, 0.0195)	0.00915 (0.00611, 0.0122)
Poor	0.00705 (0.00532, 0.00878)	0.00390 (0.00258, 0.00522)
**Adjusted^d^**		

**Gender**	**Females (n = 15563)**	**Males (n = 16236)**

Excellent	–0.0287 (–0.0376, –0.0198)	–0.0163 (–0.0263, –0.00621)
Very good	–0.00676 (–0.00887, –0.00465)	–0.00241 (–0.00390, –0.000914)
Good	0.0181 (0.0124, 0.0237)	0.0115 (0.00439, 0.0186)
Fair	0.0121 (0.00837, 0.0159)	0.00509 (0.00192, 0.00826)
Poor	0.00525 (0.00358, 0.00693)	0.00210 (0.000803, 0.00339)

^a^ Table entries are average marginal effects and 95% confidence intervals for the associations between the number of decades of pre-migration armed conflict and post-migration health status. For each individual, the number of decades of pre-migration armed conflict includes armed conflicts that occurred in their country of birth, during or after their year of birth, and before or during their year of immigration to the United States. Average marginal effects and 95% confidence intervals are estimated using sample weights. ^b^ Average marginal effects are from ordered probit regressions of the distribution of health status on the number of decades of pre-migration armed conflict. ^c^ 95% confidence intervals are computed using the delta method. ^d^ Adjusted average marginal effects and 95% confidence intervals are conditioned on the observed distributions of age, age-squared, and age-cubed.

**Table 7:** Decades Since Pre-migration Armed Conflict and Central American Immigrant Health^a^.

Health	Average Marginal Effect^b^ (95% Confidence Interval^c^)	

**Unadjusted**		

**Gender**	**Females (n = 14144)**	**Males (n = 14994)**

Excellent	–0.0430 (–0.0514, –0.0346)	–0.0494 (–0.0599, –0.0388)
Very good	–0.0118 (–0.0144, –0.00915)	–0.00861 (–0.0109, –0.00630)
Good	0.0274 (0.0221, 0.0327)	0.0354 (0.0277, 0.0430)
Fair	0.0192 (0.0152, 0.0233)	0.0161 (0.0125, 0.0197)
Poor	0.00815 (0.00623, 0.0101)	0.00648 (0.00471, 0.00825)

**Adjusted^d^**		

**Gender**	**Females (n = 14144)**	**Males (n = 14994)**

Excellent	0.0146 (0.00446, 0.0247)	–0.00103 (–0.0132, 0.0112)
Very good	0.00393 (0.00124, 0.00661)	–0.000176 (–0.00228, 0.00193)
Good	–0.00929 (–0.0158, –0.00283)	0.000736 (–0.00802, 0.00949)
Fair	–0.00649 (–0.0110, –0.00200)	0.000334 (–0.00364, 0.00430)
Poor	–0.00271 (–0.00457, –0.000846)	0.000133 (–0.00145, 0.00171)

^a^ Table entries are average marginal effects and 95% confidence intervals for the associations between the number of decades since the last pre-migration armed conflict and post-migration health status. For each individual, the last pre-migration armed conflict occurred in their country of birth, during or after their year of birth, and before or during their year of immigration to the United States. Average marginal effects and 95% confidence intervals are estimated using sample weights. ^b^ Average marginal effects are from ordered probit regressions of the distribution of health status on decades since pre-migration armed conflict. ^c^ 95% confidence intervals are computed using the delta method. ^d^ Adjusted average marginal effects and 95% confidence intervals are conditioned on the observed distributions of age, age-squared, and age-cubed.

Table 5 reports the incremental effects of the dichotomous indicator for any pre-migration armed conflict exposure on the distribution of post-migration health status, conditioned on the observed distributions of age, age squared, and age cubed, and stratified by gender. For females and males, we found negative associations between pre-migration armed conflict and post-migration health outcomes. Pre-migration armed conflict was associated with an 8.6 percentage point decrease in the probability of excellent post-migration health (95% confidence interval, 6.0 to 11.1) for females, and a 7.3 percentage point decrease in the probability of excellent post-migration health (95% confidence interval, 4.0 to 10.7) for males.

Table 6 reports the average marginal effects of the continuous variable for years of pre-migration armed conflict exposure, which we measured in decades. After adjusting for age, age squared, and age cubed, we found negative associations between the number of decades of pre-migration armed conflict exposure and post-migration health outcomes for females and males. Each additional decade of pre-migration armed conflict was associated with a 2.9 percentage point decrease in the probability of excellent post-migration health for females (95% confidence interval, 2.0 to 3.8) and a 1.6 percentage point decrease for males (95% confidence interval, 0.6 to 2.6).

Table 7 reports the age-adjusted associations between the number of decades since the most recent pre-migration armed conflict among the subgroup of individuals in the study sample with at least one year of pre-migration armed conflict, and the distribution of post-migration health status, stratified by gender. Conditional on the observed distributions of age, age squared, and age cubed, we found positive associations between the number of decades since the last conflict and post-migration health status for females, but not for males. Among the 14,144 females in the study sample who experienced at least one year of pre-migration armed conflict, each additional decade since the most recent pre-migration armed conflict is associated with a 1.5 percentage point increase in the probability of excellent post-migration health status (95% confidence interval, 0.4 to 2.5). Among the 14,994 males in the study sample who experienced at least one year of pre-migration armed conflict, we found no evidence of an association between the time since the most recent armed conflict and post-migration health status, as the average marginal effect of decades since last conflict on health was not statistically significantly different from 0 for males (95% confidence interval, –0.001 to 0.002).

## Discussion

Overall, we found that pre-migration exposure to armed conflict in Central America is negatively associated with post-migration health status in the United States. Using a dichotomous indicator for any pre-migration armed conflict, and a continuous variable for years of pre-migration armed conflict, we found that these results are statistically significant after adjusting for individual age differences. The post-migration health effects of pre-migration armed conflict were similar for males and females overall. This finding is consistent with the results of Li and Wen [27: p487], who find health effects of armed conflicts on females and males using a global cross-national time-series dataset, and is also consistent with the results of Joly and Wheaton [35: p98], who find health effects of pre-migration armed conflicts on females and males using a Canadian dataset. We did find evidence of gender differences in the duration of the effects. The health effects of armed conflict faded over time for females in our study sample, while the health effects of armed conflict persisted over time for males.

While our study extends the literature on health effects of armed conflict to include post-migration outcomes, the research design of our study does come with several caveats and limitations. Our data on country and year of birth, and year of immigration to the United States, oversimplify the temporal ordering of locations within the life course of each individual, as some individuals in our sample may have lived in multiple countries between residing in their country of birth and immigrating to the United States. Our data is also limited by its outcome measure of self-reported health status, which does not differentiate between the mental health and physical health components of health effects that can result from armed conflicts.

Further research into the post-migration health effects of pre-migration armed conflict might help to address some of the limitations inherent in our study design given the data available for analysis. Researchers should collect more data on the temporal ordering of different locations that individuals live between their country of birth and their country of destination. More studies on the post-migration health effects of pre-migration armed conflict are needed to separately measure the mental health effects and the physical health effects of different pre-migration exposures including but not limited to armed conflict. The general research design employed in this study can be expanded to study armed conflicts in regions other than Central America and to study other pre-migration exposures.
